# Identification and characterisation of clinically distinct subgroups of adults hospitalised with influenza in the USA: a repeated cross-sectional study

**DOI:** 10.1016/j.eclinm.2025.103207

**Published:** 2025-04-18

**Authors:** Catherine H. Bozio, Svetlana Masalovich, Alissa O'Halloran, Pam Daily Kirley, Cora Hoover, Nisha B. Alden, Elizabeth Austin, James Meek, Kimberly Yousey-Hindes, Kyle P. Openo, Lucy S. Witt, Maya L. Monroe, Anna Falkowski, Lauren Leegwater, Ruth Lynfield, Melissa McMahon, Daniel M. Sosin, Sarah A. Khanlian, Bridget J. Anderson, Nancy Spina, Christina B. Felsen, Maria A. Gaitan, Krista Lung, Eli Shiltz, Ann Thomas, William Schaffner, H. Keipp Talbot, Emma Mendez, Holly Staten, Carrie Reed, Shikha Garg

**Affiliations:** a Influenza Division, National Center for Immunization and Respiratory Diseases, Centers for Disease Control and Prevention, Atlanta, GA, USA; b California Emerging Infections Program, Oakland, CA, USA; c California Department of Public Health, Richmond, CA, USA; d Colorado Department of Public Health and Environment, Denver, CO, USA; e Connecticut Emerging Infections Program, Yale School of Public Health, New Haven, CT, USA; f Georgia Emerging Infections Program, Georgia Department of Public Health, Atlanta, GA, USA; g Atlanta Veterans Affairs Medical Center, Decatur, GA, USA; h Division of Infectious Diseases, Emory University School of Medicine, Atlanta, GA, USA; i Maryland Department of Health, Baltimore, MD, USA; j Michigan Department of Health and Human Services, Lansing, MI, USA; k Minnesota Department of Health, St. Paul, MN, USA; l New Mexico Department of Health, Santa Fe, NM, USA; m New Mexico Emerging Infections Program, Albuquerque, NM, USA; n New York State Department of Health, Albany, NY, USA; o University of Rochester School of Medicine and Dentistry, Rochester, NY, USA; p Ohio Department of Health, Columbus, OH, USA; q Public Health Division, Oregon Health Authority, Portland, OR, USA; r Vanderbilt University Medical Center, Nashville, TN, USA; s Salt Lake County Health Department, Salt Lake City, UT, USA

**Keywords:** Influenza, Hospitalization, Disease severity, Characterization, Latent class analysis

## Abstract

**Background:**

Patients hospitalised with influenza have heterogeneous clinical presentations and disease severity, which may complicate epidemiologic study design or interpretation. We applied latent class analysis to identify clinically distinct subgroups of adults hospitalised with influenza.

**Methods:**

We analysed cross-sectional study data on adults (≥18 years) hospitalised with laboratory-confirmed influenza from the population-based U.S. Influenza Hospitalization Surveillance Network (FluSurv-NET) including 13 states during 2017–2018 and 2018–2019 influenza seasons (October 1 through April 30). Adults were included if they were residents of the FluSurv-NET catchment area, hospitalised with laboratory-confirmed influenza during these two seasons, and had both the main case report form and the supplemental disease severity case report form completed. We constructed a latent class model to identify subgroups from multiple observed variables including baseline characteristics (age and comorbidities) and clinical course (symptoms at admission, respiratory support requirement, and development of new complications and exacerbations of underlying conditions).

**Findings:**

Among the 43,811 influenza-associated hospitalizations reported during the 2017–2018 and 2018–2019 influenza seasons, 15,873 (36.2%) were included in our analytic population: among them, 7069 (44.5%) were male and 8804 (55.5%) were female. We identified five subgroups. Subgroup A included persons of all ages with few comorbidities and 87.9% (255/290) of pregnant women. Subgroup B included older adults with comorbidities (cardiovascular disease (79.7% [3650/4581]) and diabetes (50.6% [2320/4581])). Almost all patients in subgroups C and D had asthma or chronic lung disease and high proportions with exacerbations of underlying conditions (59.7% [889/1489] and 65.1% [2274/3496], respectively). Subgroup E had the highest proportion with new complications (90.3% [1383/1531]). Subgroups D and E had the highest proportions with severe disease indicators: 21.0% (733/3496) and 50.4% (771/1531) required ICU admission, 7.2% (253/3496) and 28.0% (428/1531) required invasive mechanical ventilation, and 3.3% (116/3496) and 11.4% (174/1531) died in-hospital, respectively.

**Interpretation:**

The five identified subgroups of adults hospitalised with influenza had varying distributions of age, comorbid conditions, and clinical courses characterized by new complications versus exacerbations of existing conditions. Stratifying by these subgroups may strengthen analyses that assess the impact of influenza vaccination and antiviral treatment on risk of severe disease. Limitations included that results were based on a convenience sample within FluSurv-NET sites and were likely not representative of all adults hospitalised with influenza in the United States. Influenza testing was also clinician-driven, likely leading to under-ascertainment.

**Funding:**

10.13039/100000030Centers for Disease Control and Prevention.

**Research in context**

**Evidence before this study**

We conducted a PubMed search of the literature for articles published between January 2009 through January 2023 in English language, at the time the manuscript was first drafted. We used search terms including “influenza hospitalizations” and “heterogeneity”, “clinical phenotypes”, “subgroups”, or “disease severity”. We also searched for manuscripts that also used latent class analysis, even if applied for a different content area. Excluding reviews and editorials, we did not find a publication within that time frame that had aimed to identify subgroups of adults hospitalized with laboratory-confirmed influenza virus infection. However, a manuscript was published in February 2023 using k-medoids clustering of hospital admission characteristics (including vital signs and select clinical laboratory values) to classify severity of influenza virus infection. Given that background, we aimed to identify clinically distinct subgroups of adults hospitalized with laboratory-confirmed influenza.

**Added value of this study**

This study aimed to apply latent class analysis to identify clinically distinct subgroups of adults hospitalized with laboratory-confirmed influenza. The five subgroups had varying distributions of age and underlying conditions. Two subgroups, composed of 1) older persons with underlying respiratory conditions and a high proportion experiencing exacerbations of these conditions and 2) persons of all ages without underlying conditions and with new influenza-associated complications, had the highest frequency of severe outcomes during hospitalization.

**Implications of all the available evidence**

The heterogeneity observed in the identified subgroups may complicate our understanding of the effects of interventions such as influenza vaccines or antiviral treatment. Stratifying by these subgroups may strengthen analyses that assess the impact of influenza vaccination and antiviral treatment on risk of severe influenza disease.

## Introduction

The annual burden of influenza-associated hospitalizations is substantial, accounting for 100,000–710,000 hospitalizations and 4900–52,000 deaths,^1^ though disease severity of patients hospitalised with influenza is highly variable, in part because of differential thresholds and criteria for admitting patients who present with influenza based on baseline health. For example, otherwise healthy adults may only be hospitalised with severe influenza. In contrast, adults at higher risk for influenza-associated complications based on age or comorbidities^2^, ^3^, ^4^, ^5^ may be hospitalised with mild to severe influenza; in these patients, secondary complications, such as acute exacerbations of underlying medical conditions, may be the primary reason for admission. Other potential reasons for admission, such as for closer monitoring of patients who are frail or socially disadvantaged, or elective admission for procedures with incidental diagnosis of influenza, further complicate the ability to characterize disease severity and factors associated with severity among patients hospitalised with influenza.

Influenza prevention and control measures, such as vaccination and antiviral treatment, have been shown to attenuate severe disease.^6^, ^7^, ^8^ These modalities may differentially attenuate direct complications of influenza virus infection (e.g., acute respiratory distress syndrome (ARDS)) versus secondary complications of influenza (e.g., exacerbations of underlying conditions). Thus, evaluations of attenuation of severe disease from the influenza vaccine or antiviral treatment could be impacted by these differential and complicated reasons for hospitalization.^9^

Given the complexity in ascertaining influenza-associated disease severity amid heterogeneous clinical presentations, our primary objective was to apply latent class analysis to identify clinically distinct subgroups of adults hospitalised with laboratory-confirmed influenza during the 2017–18 and 2018–2019 seasons in 13 U.S. states.

## Methods

### Study population and data collection

We analysed cross-sectional study data from the population-based U.S. Influenza Hospitalization Surveillance Network (FluSurv-NET). The catchment area of FluSurv-NET includes select counties in the following U.S. states: California, Colorado, Connecticut, Georgia, Maryland, Michigan, Minnesota, New Mexico, New York, Ohio, Oregon, Tennessee, and Utah and captures approximately 9% of the U.S. population.^10^ Among persons residing in the catchment area and hospitalised, we defined laboratory-confirmed influenza virus infection within 14 days before or within 3 days after hospital admission, based on a positive result of molecular assay, rapid antigen test, fluorescent antibody test, or viral culture. Influenza virus testing was ordered at the discretion of the clinician.

Trained surveillance staff completed a standardized case report form for an age- and-site-stratified random sample of FluSurv-NET cases to collect information on demographic characteristics, clinical course, and outcomes during hospitalization; details about the FluSurv-NET sampling scheme has been added to the supplemental methods and also previously described.^11^, ^12^, ^13^ For the 2017–2018 and 2018–2019 seasons, a supplemental disease severity case report form was created to collect information on vital signs, selected laboratory values, use of non-invasive respiratory support, and administration of vasopressor medications, as previously described.^14^ Surveillance sites were given the option to collect the supplemental disease severity data on all or some patients within the surveillance catchment area^14^; because the approach varied across sites, cases who had this form completed were treated as a convenience sample (Supplemental Methods). Although FluSurv-NET cases were sampled to have the main case report form completed, cases in this study were not sampled and represented a convenience sample of overall sampled FluSurv-NET cases that included those with a supplemental disease severity form completed.

Adults aged ≥18 years were included in this analysis if they were residents of the FluSurv-NET catchment area, hospitalised with laboratory-confirmed influenza during the 2017–2018 and 2018–2019 influenza seasons (October 1, 2017–April 30, 2018 and October 1, 2018–April 30, 2019, respectively), and had both the main case report form and the supplemental disease severity case report form completed.

### Definitions of inputs included in the latent class model

Recognizing that people can be hospitalised for a variety of reasons, we aimed to disentangle the heterogeneous clinical presentations and identify clinically distinct subgroups of adults hospitalised with influenza. Thus, we *a priori* included variables in the latent class model that might differentiate subgroups. The latent class model included the influenza season (2017–18 or 2018–19), baseline patient characteristics (age and presence of select underlying conditions), the presence of select severe symptoms at admission (shortness of breath, seizures, or altered mental status), interventions (non-invasive mechanical ventilation [including non-home use of continuous positive airway pressure, non-home use of bilevel positive airway pressure, or high-flow nasal cannula (collected during the 2017–18 season)] or invasive mechanical ventilation) and clinical course during hospitalization (diagnoses of new complications versus exacerbations of underlying conditions) (Supplemental Table S1).

We identified and categorized new complications and exacerbations of underlying conditions through discharge diagnoses recorded on the hospital discharge summary. New complications included pneumonia, sepsis, bacteremia, acute respiratory distress syndrome, acute encephalopathy/encephalitis, acute myocarditis, and rhabdomyolysis. Exacerbations of existing underlying conditions included an exacerbation of asthma or chronic obstructive pulmonary disease (COPD) and diabetic ketoacidosis. For select discharge diagnoses, the clinical evolution was less clear, so we created two variables, one for probable new complications (including in the event of an undiagnosed underlying condition) and the other for probable exacerbations of a pre-existing condition. For the probable new complications, we included acute myocardial infarction in those without any documented record of underlying coronary artery disease, congestive heart failure exacerbation in those without underlying congestive heart failure or cardiomyopathy, stroke in those without a history of stroke, seizures in those without a history of seizure disorder, acute kidney injury in those without history of chronic kidney disease, and acute respiratory failure in those without history of chronic lung disease. For the probable exacerbations of a pre-existing condition, we included the same discharge diagnoses as the probable new complications group, but among those with a documented history of the respective condition.

### Latent class analysis

To identify subgroups of adults hospitalised with influenza, we used latent class analysis, which is a statistical technique conducted to identify latent classes or subgroups inferred from multiple observed variables using a model.^15^ A key assumption is that the observed variables are independent of each other conditional on the latent variable.^16^^,^^17^ The latent class model estimates two parameters: the latent class probability and the item-response probability. The latent class probability reflects the probability of being in a class (e.g., the proportion of patients in subgroup C). The item-response probability reflects the conditional probability of having a certain level of a variable among a specific latent class^18^ (e.g., among subgroup C, the probability of having asthma) and can be used to describe each latent class. We selected the number of latent classes based on the model with the lowest Bayesian Information Criterion and latent class interpretation, meaning the subjective label given to a latent class based on the collective interpretation of the item-response probabilities.

For the latent class analysis, people were probabilistically classified into the latent classes (subgroups) identified, and for the description of the subgroups and regression analysis, each person was classified 100% into the class that had the highest posterior probability. Once persons were deterministically classified into subgroups, additional demographic and clinical characteristics that were not included in the latent class model were examined to further describe each subgroup.

### Risk ratios of indicators of severe disease across subgroups

Once the subgroups were identified as our primary objective, we compared the risk of severe disease across subgroups to assess whether the risk was differential, which could be one indication that the subgroups were clinically distinct. We used multiple pre-specified indicators of severe disease: presence of an arterial pH value, non-invasive mechanical ventilation, invasive mechanical ventilation, vasopressor use, admission to the intensive care unit (ICU), and in-hospital death.

We first multiply imputed missing observations for influenza A subtype, race/ethnicity, receipt of influenza vaccine, and indicators of severe disease using the fully conditional specification method.^19^ The imputation model included all indicators of severe disease, covariates included in the latent class model, presence of ≥1 comorbidity, site, and month (Supplemental Methods). Using replicability of standard error estimates as a criterion for defining the number of imputations,^20^ we imputed 45 datasets. Then, in each imputed dataset, we fit a logistic regression model and used the estimated regression coefficients to estimate the relative risk of each indicator of severe disease between subgroups using the SAS macro nlmeans.^21^ Risk ratios were adjusted for age group, sex, race/ethnicity, influenza season, receipt of that season's influenza vaccine, and influenza type and A subtype. Finally, the risk ratio and standard error estimates were summarized across all imputed datasets using Rubin's rule.^22^ All analyses were performed using SAS version 9.4 (SAS Institute, Cary, NC, USA) and proc LCA.^23^^,^^24^

FluSurv-NET sites obtained human individuals and ethics approvals from their respective state health department and academic partner Institutional Review Boards (IRBs) as needed. CDC determined this activity met the requirement for public health surveillance; therefore, CDC IRB approval and informed consent were not required. Study findings are reported following the Strengthening the Reporting of Observational Studies in Epidemiology (STROBE) reporting guidelines.

### Role of the funding source

CHB, SM, AO, CR, and SG are employees of CDC and had a role in the design and conduct of the study; collection, management, analysis, and interpretation of the data; preparation, review, and approval of the manuscript; and decision to submit the manuscript for publication. CHB had full access to the data in the study, and CHB, CR, and SG had final responsibility for the decision to submit for publication.

## Results

**Fig. 1:**
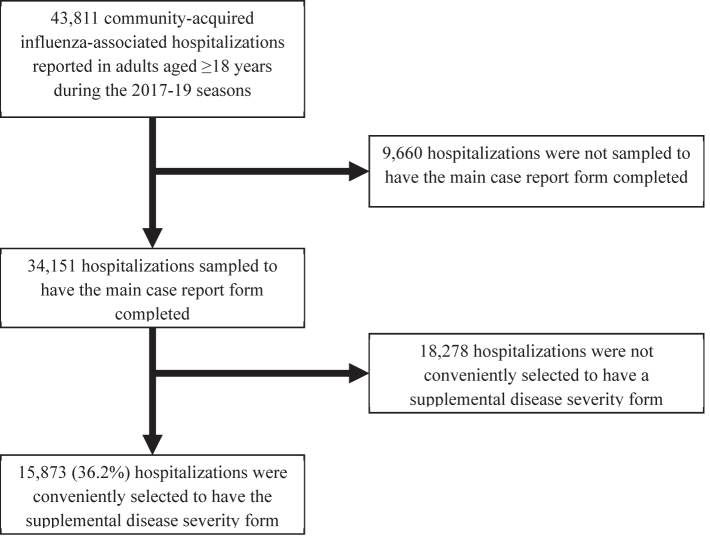
**Flow chart of inclusion and exclusion criteria for the analytic population**.

**Fig. 2:**
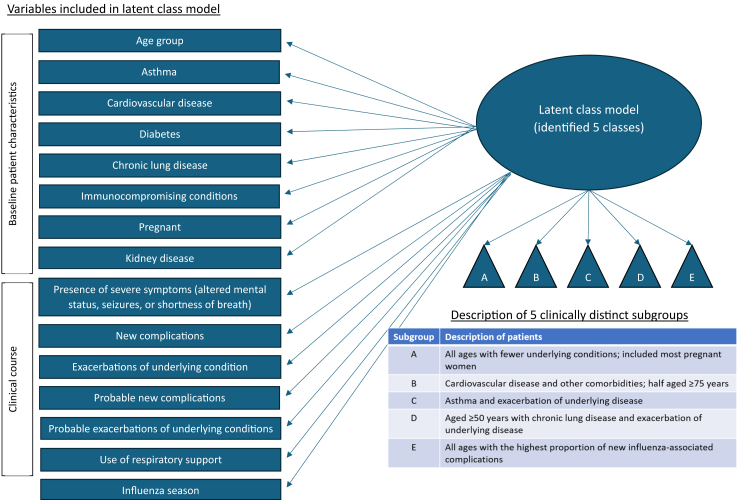
**Schematic showing the variables included in the latent class model and descriptions of the five identified subgroups**.

Among the 43,811 influenza-associated hospitalizations reported during the 2017–2018 and 2018–2019 influenza seasons, 15,873 (36.2%) were included in our analytic population (Fig. 1). Minimal differences were observed when comparing characteristics of included versus excluded patients (Supplemental Table S2). Among the 15,873 included cases, 7069 (44.5%) were men, 8988 (56.6%) were aged ≥65 years, 10,023 (63.1%) were non-Hispanic White, and 14,142 (89.1%) had one or more underlying comorbidities (Table 1). Based on our latent class model, five latent classes (subgroups) were identified, with patients probabilistically classified in subgroups using baseline patient characteristics and data on clinical course (Fig. 2 and Supplemental Table S1). After the 15,873 patients were deterministically classified, 4776 (30.1%) of patients were in subgroup A, 4581 (28.9%) in subgroup B, 1489 (9.4%) in subgroup C, 3496 (22.0%) in subgroup D, and 1531 (9.6%) in subgroup E (Table 1).

**Table 1:** Characteristics of adults aged ≥18 years hospitalised with laboratory-confirmed influenza during the 2017–18 and 2018–19 season, FluSurv-NET, overall and by latent class.

	Latent class A (N = 4776) (row %: 30.1%)	Latent class B (N = 4581) (row %: 28.9%)	Latent class C (N = 1489) (row %: 9.4%)	Latent class D (N = 3496) (row %: 22.0%)	Latent class E (N = 1531) (row %: 9.6%)	Total (N = 15,873) (row %: 100%)
	n (col %)	n (col %)	n (col %)	n (col %)	n (col %)	n (col %)
Male sex	1976 (41.4)	2321 (50.7)	460 (30.9)	1518 (43.4)	794 (51.9)	7069 (44.5)
Female sex	2800 (58.6)	2260 (49.3)	1029 (69.1)	1978 (56.6)	737 (48.1)	8804 (55.5)
Age, median (IQR)	61 (44–77)	76 (66–85)	51 (38–61)	69 (61–77)	66 (54–78)	68 (55–79)
Age						
18–49 years	1532 (32.1)	203 (4.4)	698 (46.9)	82 (2.4)	288 (18.8)	2803 (17.7)
50–64 years	1148 (24.0)	796 (17.4)	523 (35.1)	1187 (34.0)	428 (28.0)	4082 (25.7)
65–74 years	754 (15.8)	1050 (23.0)	185 (12.4)	1128 (32.3)	347 (22.7)	3464 (21.8)
≥75 years	1342 (28.1)	2532 (55.3)	83 (5.6)	1099 (31.4)	468 (30.6)	5524 (34.8)
Race/Ethnicity						
Non-Hispanic white	2896 (60.6)	2929 (63.9)	697 (46.8)	2497 (71.4)	1004 (65.6)	10,023 (63.1)
Non-Hispanic black	1097 (23.0)	1105 (24.1)	561 (37.7)	699 (20.0)	251 (16.4)	3713 (23.4)
Hispanic	431 (9.0)	250 (5.5)	134 (9.0)	141 (4.0)	138 (9.0)	1094 (6.9)
Other^a^	170 (3.6)	154 (3.4)	50 (3.4)	57 (1.6)	86 (5.6)	517 (3.3)
Missing	182 (3.8)	143 (3.1)	47 (3.2)	102 (2.9)	52 (3.4)	526 (3.3)
Smoker						
Current	883 (18.5)	504 (11.0)	489 (32.8)	1431 (41.0)	332 (21.7)	3640 (22.9)
Former	958 (20.1)	1655 (36.1)	302 (20.3)	1507 (43.1)	394 (25.7)	4816 (30.3)
No/Unknown	2935 (61.4)	2422 (52.9)	698 (46.9)	557 (15.9)	805 (52.6)	7417 (46.7)
Residence						
Nursing home	514 (10.8)	894 (19.5)	47 (3.2)	441 (12.6)	253 (16.5)	2149 (13.5)
Congregate	159 (3.3)	61 (1.3)	70 (4.7)	92 (2.6)	44 (2.9)	426 (2.7)
Private residence	4050 (84.8)	3606 (78.7)	1364 (91.6)	2948 (84.3)	1230 (80.3)	13,198 (83.2)
Other	53 (1.1)	20 (0.4)	8 (0.5)	15 (0.4)	4 (0.3)	100 (0.6)
No underlying conditions	1465 (30.7)	10 (0.2)	16 (1.1)	3 (0.1)	237 (15.5)	1731 (10.9)
Asthma	339 (7.1)	473 (10.3)	1416 (95.1)	910 (26.0)	145 (9.5)	3283 (20.7)
Chronic lung disease^b^	83 (1.7)	532 (11.6)	168 (11.3)	3459 (98.9)	24 (1.6)	4266 (26.9)
Diabetes	911 (19.1)	2320 (50.6)	368 (24.7)	1099 (31.4)	489 (31.9)	5187 (32.7)
Cardiovascular disease^c^	48 (1.0)	3650 (79.7)	124 (8.3)	1756 (50.2)	473 (30.9)	6051 (38.1)
Blood disorders/hemoglobinopathy	150 (3.1)	100 (2.2)	32 (2.2)	63 (1.8)	22 (1.4)	367 (2.3)
Neurologic disorder	831 (17.4)	1135 (24.8)	152 (10.2)	546 (15.6)	320 (20.9)	2984 (18.8)
Immunocompromising condition^d^	389 (8.1)	414 (9.0)	16 (1.1)	185 (5.3)	135 (8.8)	1139 (7.2)
Kidney disease^e^	92 (1.9)	2125 (46.4)	29 (2.0)	607 (17.4)	179 (11.7)	3032 (19.1)
Liver disease	230 (4.8)	221 (4.8)	84 (5.6)	237 (6.8)	93 (6.1)	865 (5.5)
Obesity	1560 (32.7)	1712 (37.4)	843 (56.6)	1398 (40.0)	628 (41.0)	6141 (38.7)
Pregnancy during hospitalization	255 (5.3)	0 (0.0)	34 (2.3)	0 (0.0)	1 (0.1)	290 (21.7)
Receipt of current season's influenza vaccine^f^						
Yes	1937 (40.6)	2561 (55.9)	594 (39.9)	1929 (55.2)	600 (39.2)	7621 (48.0)
No	1988 (41.6)	1312 (28.6)	684 (45.9)	1101 (31.5)	627 (41.0)	5712 (36.0)
Unknown	851 (17.8)	708 (15.5)	211 (14.2)	466 (13.3)	304 (20.0)	2540 (16.0)
Received antiviral treatment within two weeks before admission or any time during hospitalization						
Yes	2688 (56.3)	2602 (56.8)	789 (53.0)	1829 (52.3)	727 (47.5)	8635 (54.4)
No	2035 (42.6)	1963 (42.9)	699 (46.9)	1663 (47.5)	801 (52.3)	7161 (45.1)
Missing	53 (1.1)	16 (0.4)	1 (0.1)	4 (0.1)	3 (0.2)	77 (0.5)
Presence of severe symptoms^g^	2250 (47.1)	3046 (66.5)	1388 (93.2)	3274 (93.7)	1359 (88.8)	11,317 (71.3)
Use of respiratory support^h^	75 (1.6)	357 (7.8)	159 (10.7)	927 (26.5)	838 (54.7)	2356 (14.8)
Hospital length of stay, median (IQR)	3 (2–4)	4 (2–6)	3 (2–4)	4 (2–7)	6 (3–11)	3 (2–6)

a Other race is defined as Asian/Pacific Islander, American Indian/Alaskan Native, Multiracial, and other races not listed.

b Chronic lung disease was defined as having as chronic obstructive pulmonary disease (COPD) or chronic bronchitis.

c Cardiovascular disease was defined as having atherosclerotic cardiovascular disease, cerebral vascular incident/stroke, coronary artery disease, ischemic or non-ischemic cardiomyopathy, or heart failure.

d Immunocompromising conditions were defined as having cancer (current, in treatment, or diagnosed within the last 12 months) or bone marrow or organ transplant.

e Kidney disease was defined as having chronic kidney disease/chronic renal insufficiency or end-stage renal disease.

f Trained surveillance staff ascertained a patient's current season influenza vaccination status by reviewing up to four sources (hospital medical record, state immunization registry or immunization information system, follow-up with an outpatient primary care provider or long-term care facility, or interview of the patient or a proxy). A patient was considered vaccination if documentation in at least one source indicated receipt of at least one dose of the current season's influenza vaccine at least 2 weeks before a positive influenza test date. The patient was considered unvaccinated if documentation in at least one source indicated no receipt of the current season's influenza vaccine.

g Presence of severe symptoms was defined as having either shortness of breath, seizures, or altered mental status as a self-reported sign/symptom at the time of hospital admission.

h Respiratory support included the use of either non-invasive or invasive mechanical ventilation during the hospitalization.

Fig. 2 specifies the variables included into the latent class model and provides brief descriptions for each subgroup. Subgroup A was comprised of 4776 patients of all ages, and 1465 (30.7%) had no underlying conditions (the highest proportion across all classes) (Table 1). Among the 290 pregnant women included in this analysis, 255 (87.9%) were classified in subgroup A. Similarly, among patients aged 18–49 years and with no underlying conditions, 54.7% (1532/2803) and 84.6% (1465/1731) were classified in subgroup A, respectively (Supplemental Table S3). Subgroup B was comprised of predominantly older adults (55.3% [2532/4581] aged ≥75 years); 79.7% (3650/4581) and 50.6% (2320/4581) of patients in this subgroup had cardiovascular disease and diabetes, respectively, but kidney disease (46.4%, 2125/4581) and obesity (37.4%, 1712/4581) were also common (Table 1). Among 5664 adults hospitalised with influenza who had ≥3 comorbidities, almost half (2543 or 44.9%) were classified into subgroup B (Supplemental Table S3).

In subgroup C, almost all (95.1%, 1416/1489) patients had asthma. This subgroup also had the highest proportions of 18–49-year-old patients (46.9%, 698/1489) and non-Hispanic Black persons (37.7%, 561/1489) compared to other subgroups (range: 2.4% [82/3496] - 32.1% [1532/4776] and 16.4% [251/1531] - 24.1% [1105/4581], respectively) (Table 1). Subgroup C also had the highest proportion of female patients (69.1%, 1029/1489) compared to other subgroups (range: 48.1% [737/1531]—58.6% [2800/4776]). Subgroup D was almost entirely comprised of patients with chronic lung disease (98.9%, 3459/3496). Almost all (97.6%, 3414/3496) patients in this subgroup were aged ≥50 years, and 50.2% (1756/3496) also had cardiovascular disease. Subgroup E was comprised of 1531 patients of all ages with some underlying conditions; the three most common were obesity (41.0%, 628), cardiovascular disease (30.9%, 473), and diabetes (31.9%, 489).

Frequencies of new complications and exacerbations of underlying conditions differed across subgroups (Table 2). Compared with the other subgroups, new and probable new complications were most common in subgroup E: pneumonia (58.3%, 893/1531), sepsis (47.8%, 731/1531), acute respiratory distress syndrome (9.1%, 140/1531), encephalopathy/encephalitis (15.5%, 237/1531), acute kidney injury (36.3%, 556/1531) and acute respiratory failure (68.6%, 1050/1531). Exacerbations of underlying conditions were most common in subgroups C and D. Within subgroup C, 49.1% (731/1489) of patients had an exacerbation of underlying asthma. When limited to asthmatic patients within each subgroup, subgroup C had the highest proportion of asthma exacerbations (51.3%, 726/1416) compared to other subgroups (range: 0% [0/339] −13.0% [118/910] (Supplemental Figure S1A). In subgroup D, 62.6% (2190/3496) had an exacerbation of underlying COPD and 37.2% (1299/3496) had acute respiratory failure in the setting of chronic lung disease. When limited to patients with chronic lung disease within each subgroup, subgroup D had the highest proportion of COPD exacerbations (62.7%, 2169/3459) compared to other subgroups (range: 0% [0/83] - 35.1% [59/168]) (Supplemental Figure S1B). While exacerbations of underlying conditions were rare in subgroup A and B, acute kidney injury in those with chronic kidney disease (12.2%, 560/4581) was most common in subgroup B.

**Table 2:** Frequency of new or probable new complications and exacerbations or probable exacerbations of underlying conditions, overall and by latent class.

	Latent class A (N = 4776)	Latent class B (N = 4581)	Latent class C (N = 1489)	Latent class D (N = 3496)	Latent class E (N = 1531)	Total (N = 15,873)
	n (col %)	n (col %)	n (col %)	n (col %)	n (col %)	n (col %)
New complication^a^						
Any new complication^b^	1261 (26.4)	1301 (28.4)	301 (20.2)	1200 (34.3)	1383 (90.3)	5446 (34.3)
Pneumonia	780 (16.3)	786 (17.2)	188 (12.6)	803 (23.0)	893 (58.3)	3450 (21.7)
Sepsis	525 (11.0)	493 (10.8)	144 (9.7)	516 (14.8)	731 (47.8)	2409 (15.2)
Bacteremia	45 (0.9)	60 (1.3)	7 (0.5)	37 (1.1)	79 (5.2)	228 (1.4)
Acute respiratory distress syndrome	6 (0.1)	9 (0.2)	7 (0.5)	33 (0.9)	140 (9.1)	195 (1.2)
Encephalopathy/encephalitis	100 (2.1)	208 (4.5)	13 (0.9)	132 (3.8)	237 (15.5)	690 (4.4)
Acute myocarditis	3 (0.1)	3 (0.1)	1 (0.1)	1 (0.0)	3 (0.2)	11 (0.1)
Rhabdomyolysis	51 (1.1)	41 (0.9)	3 (0.2)	13 (0.4)	33 (2.2)	141 (0.9)
Probable new complications occurring in patients without a history of the corresponding underlying condition^c^						
Any probable new complication^b^	645 (13.5)	855 (18.7)	294 (19.7)	344 (9.8)	1411 (92.2)	3549 (22.4)
Acute myocardial infarction	28 (0.6)	50 (1.1)	3 (0.2)	55 (1.6)	63 (4.1)	199 (1.3)
Congestive heart failure exacerbation with underlying congestive heart failure or cardiomyopathy	34 (0.7)	68 (1.5)	8 (0.5)	48 (1.4)	73 (4.8)	231 (1.5)
Stroke	14 (0.3)	21 (0.5)	2 (0.1)	5 (0.1)	21 (1.4)	63 (0.4)
Seizures	16 (0.3)	11 (0.2)	2 (0.1)	4 (0.1)	17 (1.1)	50 (0.3)
Acute kidney injury	348 (7.3)	310 (6.8)	66 (4.4)	260 (7.4)	556 (36.3)	1540 (9.7)
Acute respiratory failure	267 (5.6)	479 (10.5)	230 (15.5)	2 (0.1)	1050 (68.6)	2028 (12.8)
Exacerbations of underlying conditions^d^						
Any exacerbation of underlying conditions^b^	59 (1.2)	107 (2.3)	889 (59.7)	2274 (65.1)	76 (5.0)	3405 (21.5)
Asthma exacerbation	2 (0.0)	31 (0.7)	731 (49.1)	130 (3.7)	16 (1.1)	910 (5.7)
Chronic obstructive pulmonary disease exacerbation	15 (0.3)	54 (1.2)	130 (8.7)	2190 (62.6)	47 (3.1)	2436 (15.4)
Diabetic ketoacidosis	42 (0.9)	24 (0.5)	45 (3.0)	8 (0.2)	17 (1.1)	136 (0.9)
Probable exacerbations of underlying conditions occurring in patients with a history of the corresponding underlying condition^e^						
Any probable exacerbation of underlying conditions^b^	0 (0.0)	1093 (23.9)	21 (1.4)	1565 (44.8)	174 (11.4)	2853 (18.0)
Acute myocardial infarction	0 (0.0)	99 (2.2)	0 (0.0)	53 (1.5)	23 (1.5)	175 (1.1)
Congestive heart failure exacerbation with underlying congestive heart failure or cardiomyopathy	0 (0.0)	350 (7.6)	1 (0.1)	243 (7.0)	58 (3.8)	652 (4.1)
Stroke	0 (0.0)	20 (0.4)	0 (0.0)	6 (0.2)	4 (0.3)	30 (0.2)
Seizures	0 (0.0)	25 (0.6)	6 (0.4)	9 (0.3)	15 (1.0)	55 (0.4)
Acute kidney injury	0 (0.0)	560 (12.2)	2 (0.1)	160 (4.6)	55 (3.6)	777 (4.9)
Acute respiratory failure	0 (0.0)	168 (3.7)	12 (0.8)	1299 (37.2)	33 (2.2)	1512 (9.5)

a New complications were identified through discharge diagnoses recorded on the hospital discharge summary.

b Individual complications or exacerbations are not mutually exclusive. Patients could have experienced more than one new complication or exacerbation.

c We categorized select discharge diagnoses as probable new complications if they occurred in individuals without a history of the corresponding condition. These diagnoses included acute myocardial infarction in those without any underlying coronary artery disease, congestive heart failure exacerbation in those without underlying congestive heart failure or cardiomyopathy, stroke in those without a history of stroke, seizures in those without a history of seizure disorder, acute kidney injury in those without history of chronic kidney disease, and acute respiratory failure in those without history of chronic lung disease.

d Exacerbations of underlying conditions were defined as a discharge diagnosis of asthma exacerbation or chronic obstructive pulmonary disease (COPD) exacerbation or diabetic ketoacidosis as these conditions occurred in patients with chronic underlying asthma, COPD, or diabetes mellitus.

e We categorized select discharge diagnoses as probable exacerbations of underlying conditions if they occurred in individuals with a history of the corresponding underlying condition. These diagnoses included acute myocardial infarction in those with any underlying coronary artery disease, congestive heart failure exacerbation in those with underlying congestive heart failure or cardiomyopathy, stroke in those with a history of stroke, seizures in those with a history of seizure disorder, acute kidney injury in those with history of chronic kidney disease, and acute respiratory failure in those with history of chronic lung disease.

**Fig. 3:**
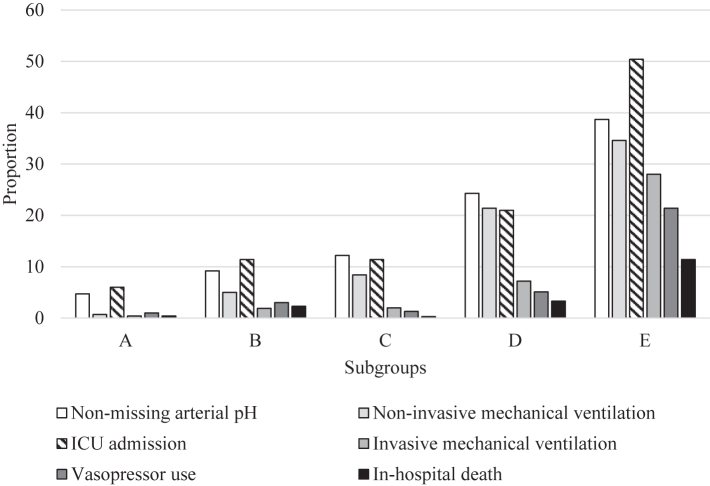
**Prevalence of each indicator of disease severity within subgroups of adults hospitalised with influenza**.

The proportion of patients with severe disease indicators varied across subgroups (Fig. 3). Within subgroup A, 6.0% (286/4776) of patients required ICU admission and less than 1% required invasive mechanical ventilation (0.4%, 19/4776) or died (0.4%, 17/4776). Within subgroup B, 11.4% (522/4581) of patients required ICU admission, 1.9% (89/4581) received invasive mechanical ventilation, and 2.3% (106/4581) died. Within subgroup C, the proportions with severe disease indicators were similar to those seen for subgroup B: 11.4% (169/1489) required ICU admission, 2.0% (29/1489) received invasive mechanical ventilation, but a lower proportion died (0.3%, 4/1489). Subgroups D and E had the highest proportions of patients with severe disease. Within subgroup D, 21.0% (733/3496) required ICU admission, 21.4% (749/3496) received non-invasive mechanical ventilation, 7.2% (253/3496) received invasive mechanical ventilation, and 3.3% (116/3496) died. Within subgroup E, half (50.4%, 771/1531) required ICU admission, 34.6% (529/1531) received non-invasive mechanical ventilation, 28.0% (428/1531) received invasive mechanical ventilation, 21.4% (327/1531) received vasopressor support, and 11.4% (174/1531) died. For each indicator of severe disease, the adjusted risk ratios (aRRs) overall differed across the subgroups, compared to subgroup A (Supplemental Table S4). Subgroups E and D consistently had the highest aRRs for all severe disease indicators.

## Discussion

Among adults hospitalised with influenza across two seasons, we identified five clinically distinct subgroups using latent class analysis. Briefly, these subgroups reflected A) patients of all ages with few underlying conditions and most pregnant women included in this analysis, B) primarily patients with cardiovascular disease and other underlying conditions, C) primarily patients with asthma and exacerbation of underlying disease, and the remaining pregnant women included in this analysis, D) primarily patients ≥50 years old with chronic lung disease and exacerbation of underlying disease, and E) patients of all ages with some underlying conditions and the highest proportion of new influenza-associated complications and severe disease indicators. Subgroups D and E also had the highest adjusted risk ratios for all indicators of severe disease. Importantly, this analysis helped us to identify subgroups that have been most impacted by seasonal and pandemic influenza viruses: patients with influenza-associated complications and patients with exacerbations of respiratory-related comorbidities.^25^^,^^26^ The heterogeneity seen here may complicate our understanding of interventions such as influenza vaccines or antiviral treatment; stratifying by these subgroups of influenza-associated hospitalizations may strengthen analyses that assess the impact of these interventions on attenuation of severe influenza-associated disease.

Influenza can cause severe disease through direct viral pathogenesis, exacerbation of a comorbidity, or a mixture of both. A high proportion of patients in subgroups C and D had exacerbations of underlying conditions, whereas a high proportion of patients in subgroup E presented with new complications, primarily acute respiratory failure, pneumonia and sepsis. Patients in subgroups C and D might reflect groups of adults experiencing indirect effects of influenza virus infection, as the influenza virus infection may have triggered an exacerbation of asthma or COPD that prompted hospitalization. Patients in subgroup E might reflect those with direct effects of influenza virus infection. In contrast, subgroups A and B included most pregnant women or patients with advanced age or multimorbidity, respectively, and otherwise did not have clear indicators for what might have triggered hospitalization; these two classes may reflect a higher proportion of patients admitted for closer monitoring rather than entirely for management of severe influenza-associated disease.

For evaluations of whether influenza vaccination or antiviral treatment attenuate influenza disease severity, it may be important to differentiate patients based on whether they are hospitalised due to direct or indirect effects of influenza.^27^ Analyzing all hospitalised patients as a single population likely muddles the effect of vaccination or antiviral treatment, which might differ across these clinically distinct groups. For example, as subgroup E had the highest proportion of patients with new influenza-associated complications, it is possible that we might observe a stronger association between antiviral treatment and attenuation of severe disease among patients in this subgroup compared with other subgroups. We plan to use these identified subgroups of influenza-associated hospitalizations for a subsequent evaluation of attenuation of severe disease and assess whether the effect of vaccination and/or antiviral treatment on the risk of severe disease is meaningfully modified based on the various clinical pathways leading to hospitalization reflected in these subgroups.

Two subgroups (C and D) were almost entirely comprised each of persons with asthma or chronic lung disease, respectively, and together made up nearly a third of the influenza-associated hospitalizations. Because not everyone with these comorbidities were in those two subgroups, that alone would make clinically operationalizing these subgroups difficult and warrants better understanding of why these subgroups had only some patients with these comorbidities. Within these two subgroups, patients with pre-existing asthma or chronic lung disease had higher proportions of exacerbations compared to patients in other subgroups with those comorbidities. Thus, patients with these comorbidities might have been distinguished into their own separate subgroups in part based on the occurrence of exacerbations.

Additionally, the subgroup of persons with asthma (subgroup C) had the highest proportion of non-Hispanic Black persons, which may reflect the higher prevalence of asthma among non-Hispanic Black persons compared to other racial/ethnic groups in the analytic population. Additionally, in subgroup C, non-Hispanic Black persons had a higher proportion of asthma exacerbation compared to non-Hispanic White persons. The higher proportion of exacerbations within this subgroup among non-Hispanic Black persons might reflect a lower threshold for admission of persons with asthma for closer monitoring and may also reflect reduced access to preventive healthcare and asthma care control and impact of social determinants of health among certain racial and ethnic groups.^28^ Elucidating why a subset of persons with these comorbidities were classified in these two subgroups will be important in interpreting results in the subsequent evaluation of attenuation of severe disease.

Our analysis had limitations. First, results were based on a convenience sample within FluSurv-NET sites and were likely not representative of all adults hospitalised with influenza in the United States. Second, influenza testing was clinician-driven, likely leading to under-ascertainment, which may have been greater for people with more atypical presentation of influenza who may not have been tested. Finally, we included variables in the latent class model and interpreted results based on variables from the existing case report form, though other variables may have also been useful to include if they had been available (such as frailty, code status, and post-discharge sequelae).

In conclusion, five clinically distinct subgroups of influenza-associated hospitalizations were identified in adults during two influenza seasons. Stratifying by these subgroups of influenza-associated hospitalizations for studies of severity attenuation may help to better understand and assess the impact of influenza vaccination and antiviral treatment. Such findings could be beneficial in communicating the benefits of annual influenza vaccination and encouraging vaccine uptake and early initiation of antiviral treatment, particularly amongst those at higher risk of influenza-associated complications, to reduce the risk of severe disease from influenza.

## Contributors

Conceptualization: CHB, CR, SG.

Data curation: CHB, AO, SG.

Formal analysis: CHB, SM.

Funding acquisition: NBA, JM, KYH, MLM, RL, DMS, BJA, AT, WS, HKT.

Investigation and methodology: CHB, CR, SG.

Supervision: CR, SG.

Writing—original draft: CHB.

Writing—review and editing: CHB, SM, AO, PDK, CH, NBA, EA, JM, KYH, KPO, LSW, MLM, AF, LL, RL, MM, DMS, SAK, BJA, NS, CBF, MAG, KL, ES, AT, WS, HKT, EM, HS, CR, SG.

CHB and SM have access to and verify the underlying study data.

## Data sharing statement

Individuals interested in receiving a limited dataset can submit a brief proposal to the corresponding author (CHB) for review and consideration by the CDC and FluSurv-NET site partners.

## Declaration of interests

RL is an Associate Editor for American Academy of Pediatrics Red Book (Report on the Committee on Infectious Diseases); fee donated to Minnesota Department of Health. ES reports being a recipient of Epidemiology and Laboratory Capacity (ELC) and Immunizations and Vaccines for Children (VFC) grant funding from CDC to support vaccine preventable disease epidemiology staffing. All other authors declare no competing interests.
