# Supplementary material for: Identification and characterisation of clinically distinct subgroups of adults hospitalised with influenza in the USA: a repeated cross-sectional study
Source: eClinicalMedicine. 2025 Apr 18;83:103207. doi: 10.1016/j.eclinm.2025.103207 (PMC12032903; doi:10.1016/j.eclinm.2025.103207)
Supplement: Supplemental Materials [file mmc1.pdf]

Supplemental Table 1. Results from latent class model aimed at identifying five subgroups[a] of adults hospitalised with laboratory-confirmed influenza during the 2017-19 seasons

Class letter:	A	B	C	D	E
Latent class probabilities:	0.283	0.307	0.095	0.209	0.106
	Item-response probabilities[b]				
Age (18-49 years)	0.336	0.049	0.453	0.023	0.175
Age (50-64 years)	0.242	0.180	0.338	0.338	0.291
Age (65-74 years)	0.153	0.235	0.125	0.319	0.228
Age (≥75 years)	0.270	0.535	0.084	0.320	0.306
Asthma	0.091	0.106	0.898	0.254	0.095
Cardiovascular disease[c]	0.043	0.713	0.095	0.515	0.319
Diabetes	0.170	0.497	0.263	0.319	0.325
Chronic lung disease[d]	0.024	0.137	0.152	0.972	0.022
Immunocompromising conditions[e]	0.076	0.092	0.017	0.051	0.093
Pregnant	0.056	0.000	0.024	0.000	0.001
Kidney disease[f]	0.031	0.413	0.021	0.187	0.134
Presence of severe symptoms[g]	0.487	0.683	0.898	0.930	0.868
New complications[h]	0.275	0.291	0.204	0.336	0.817
Exacerbations of underlying condition[i]	0.013	0.026	0.603	0.673	0.049
Probable new complications[j]	0.139	0.188	0.198	0.100	0.820
Probable exacerbation[k]	0.000	0.230	0.022	0.449	0.125
Use of respiratory support[l]	0.018	0.086	0.113	0.266	0.479
Influenza season (2018-2019)	0.425	0.424	0.477	0.481	0.545

[a]Five latent classes were selected based on the lowest Bayesian Information Criterion (BIC) and latent class interpretation. The BIC for 4, 5, and 6 classes were 18479.49, 16859.51, and 15486.34, respectively.

[b]Only the item-response probabilities shown reflect the conditional probability of having that level of specified variable (e.g., the probability of having asthma within the first latent class), except for age.

[c]Cardiovascular disease was defined as having atherosclerotic cardiovascular disease, cerebral vascular incident/stroke, coronary artery disease, ischemic or non-ischemic cardiomyopathy, or heart failure.

[d]Chronic lung disease was defined as having as chronic obstructive pulmonary disease (COPD) or chronic bronchitis.

[e]Immunocompromising conditions were defined as having cancer (current, in treatment, or diagnosed within the last 12 months) or bone marrow or organ transplant.

[f]Kidney disease was defined as having chronic kidney disease/chronic renal insufficiency or end-stage renal disease.

[g]Presence of severe symptoms was defined as having either shortness of breath, seizures, or altered mental status as a self-reported sign/symptom at the time of hospital admission.

[h]Complications were identified through discharge diagnoses recorded on the hospital discharge summary and included pneumonia, sepsis, bacteremia, acute respiratory distress syndrome, acute encephalopathy/encephalitis, acute myocarditis, and rhabdomyolysis.

[i]An exacerbation of an existing underlying condition was defined as a discharge diagnosis of asthma exacerbation or chronic obstructive pulmonary disease (COPD) exacerbation or diabetic ketoacidosis.

[j]We considered select discharge diagnoses that could occur through mixed mechanisms and likely could be acute complications, which included acute myocardial infarction in those without any underlying coronary artery disease, congestive heart failure exacerbation in those without underlying congestive heart failure or cardiomyopathy, stroke in those without a history of stroke, seizures in those without a history of seizure disorder, acute kidney injury in those without history of chronic kidney disease, and acute respiratory failure in those without history of chronic lung disease.

[k]We considered select discharge diagnoses that could occur through mixed mechanisms and likely could be an exacerbation of a pre-existing underlying condition, which included acute myocardial infarction in those with any underlying coronary artery disease, congestive heart failure exacerbation in those with underlying congestive heart failure or cardiomyopathy, stroke in those with a history of stroke, seizures in those with a history of seizure disorder, acute kidney injury in those with history of chronic kidney disease, and acute respiratory failure in those with history of chronic lung disease.

[l]Respiratory support included the use of either non-invasive or invasive mechanical ventilation during the hospitalization.

Supplemental Table 2. Characteristics of patients across whether they were randomly sampled to have the main case report form completed and conveniently selected by sites to have the supplemental disease severity form completed

	FluSurv-NET cases that were randomly sampled to have the main case report form completed and conveniently selected by sites to have a supplemental disease severity form completed (N=15,873) n (col%)	FluSurv-NET cases who were randomly sampled to have only the main case report form completed (N=18,278) n (col %)	FluSurv-NET cases that were not sampled to have either the main case report form or the supplemental disease severity form completed (N=9,660) n (col %)
Age*			
18-49 years	2803 (17.7)	4246 (23.2)	0 (0)
50-64 years	4082 (25.7)	5104 (27.9)	1391 (14.4)
65-74 years	3464 (21.8)	3102 (17.0)	2935 (30.4)
≥75 years	5524 (34.8)	5826 (31.9)	5334 (55.2)
Sex			
Male	7069 (44.5)	8213 (44.9)	4436 (45.9)
Female	8804 (55.5)	10065 (55.1)	5224 (54.1)
State*			
California	491 (3.1)	2561 (14.0)	27744 (28.4)
Colorado	598 (3.8)	2994 (16.4)	0 (0)
Connecticut	1156 (7.3)	1338 (7.3)	0 (0)
Georgia	188 (1.2)	2261 (12.4)	1646 (17.0)
Maryland	3968 (25.0)	271 (1.5)	0 (0)
Michigan	1757 (11.1)	505 (2.8)	0 (0)
Minnesota	998 (6.3)	2371 (13.0)	1095 (11.3)
New Mexico	421 (2.7)	631 (3.5)	719 (7.4)
New York	2205 (13.9)	2260 (12.4)	926 (9.6)
Ohio	0 (0)	1595 (8.7)	1388 (14.4)
Oregon	273 (1.7)	1230 (6.7)	1142 (11.8)
Tennessee	2417 (15.2)	181 (1.0)	0 (0)
Utah	1401 (8.8)	80 (0.4)	0 (0)
Month of admission			
October	102 (0.6)	149 (0.8)	84 (0.9)
November	267 (1.7)	462 (2.5)	209 (2.2)
December	1843 (11.6)	3089 (16.9)	1828 (18.9)
January	4494 (28.3)	5698 (31.2)	3671 (38.0)
February	4285 (27.0)	4185 (22.9)	1982 (20.5)
March	3476 (21.9)	3569 (19.5)	1440 (14.9)
April	1406 (8.9)	1126 (6.2)	446 (4.6)
Died in-hospital*			

Yes	419 (2.6)	919 (5.0)	0 (0)
No	15453 (97.4)	17243 (94.3)	9642 (99.8)
Missing	1 (0.0)	116 (0.6)	18 (0.2)

*Age and whether the patient died in-hospital were used as sampling strata. Sites were given the option to implement the random sampling scheme for the main case report form and to collect the supplemental disease severity form on all or some patients (supplemental methods).

Supplemental Table 3. Distribution of latent classes across adults hospitalised with influenza with select demographic or clinical characteristics.

	Latent class A (N=4776)	Latent class B (N=4581)	Latent class C (N=1489)	Latent class D (N=3496)	Latent class E (N=1531)	Row Total
	n (row %)					
Sex						
Male	1976 (28.0)	2321 (32.8)	460 (6.5)	1518 (21.5)	794 (11.2)	7069
Female	2800 (31.8)	2260 (25.7)	1029 (11.7)	1978 (22.5)	737 (8.4)	8804
Age						
18-49 years	1532 (54.7)	203 (7.2)	698 (24.9)	82 (2.9)	288 (10.3)	2803
50-64 years	1148 (28.1)	796 (19.5)	523 (12.8)	1187 (29.1)	428 (10.5)	4082
65-74 years	754 (21.8)	1050 (30.3)	185 (5.3)	1128 (32.6)	347 (10.0)	3464
≥75 years	1342 (24.3)	2532 (45.8)	83 (1.5)	1099 (19.9)	468 (8.5)	5524
Race/Ethnicity						
Non-Hispanic White	2896 (28.9)	2929 (29.2)	697 (7.0)	2497 (24.9)	1004 (10.0)	10023
Non-Hispanic Black	1097 (29.5)	1105 (29.8)	561 (15.1)	699 (18.8)	251 (6.8)	3713
Hispanic	431 (39.4)	250 (22.9)	134 (12.3)	141 (12.9)	138 (12.6)	1094
Other[a]	170 (32.9)	154 (29.8)	50 (9.7)	57 (11.0)	86 (16.6)	517
Missing	182 (34.6)	143 (27.2)	47 (8.9)	102 (19.4)	52 (9.9)	526
Pregnant during hospitalization	255 (87.9)	0 (0)	34 (11.7)	0 (0)	1 (0.3)	290
Number of underlying medical conditions						
0	1465 (84.6)	10 (0.6)	16 (0.9)	3 (0.2)	237 (13.7)	1731
1	2042 (50.2)	659 (16.2)	338 (8.3)	515 (12.7)	511 (12.6)	4065
2	1001 (22.7)	1369 (31.0)	630 (14.3)	942 (21.4)	471 (10.7)	4413
≥3	268 (4.7)	2543 (44.9)	505 (8.9)	2036 (36.0)	312 (5.5)	5664

[a]Other race is defined as Asian/Pacific Islander, American Indian/Alaskan Native, Multiracial, and other races not listed.

Supplemental Table 4. Unadjusted and adjusted* risk ratios (RRs) of indicators of disease severity across subgroups of adults hospitalised with influenza

	Latent classes (Number of adults)	Subgroup A (N=4776)	Subgroup B (N=4581)	Subgroup C (N=1489)	Subgroup D (N=3496)	Subgroup E (N=1531)
Non-missing arterial pH	n (col %)	222 (4.7)	422 (9.2)	182 (12.2)	849 (24.3)	592 (38.7)
	Unadjusted RR (95% CI)	ref	1.98 (1.67, 2.29)	2.63 (2.14, 3.13)	5.22 (4.48, 5.95)	8.30 (7.12, 9.49)
	Adjusted RR* (95% CI)	ref	2.29 (1.91, 2.67)	2.58 (2.08, 3.08)	5.84 (4.96, 6.73)	9.44 (7.99, 10.89)
Non-invasive mechanical ventilation	n (col %)	31 (0.7)	230 (5.0)	125 (8.4)	749 (21.4)	529 (34.6)
	Unadjusted RR (95% CI)	ref	5.01 (3.67, 6.34)	7.22 (5.13, 9.31)	18.06 (13.52, 22.60)	31.27 (23.41, 39.13)
	Adjusted RR* (95% CI)	ref	5.29 (3.84, 6.74)	7.95 (5.62, 10.28)	20.15 (14.96, 25.34)	36.26 (26.89, 45.63)
Invasive mechanical ventilation	n (col %)	19 (0.4)	89 (1.9)	29 (2.0)	253 (7.2)	428 (28.0)
	Unadjusted RR (95% CI)	ref	4.75 (2.43, 7.07)	4.82 (2.08, 7.57)	17.67 (9.57, 25.78)	67.88 (37.32, 98.44)
	Adjusted RR* (95% CI)	ref	7.30 (3.86, 10.92)	4.01 (1.73, 6.30)	25.15 (13.34, 36.97)	91.71 (49.70, 133.71)
Vasopressor use	n (col %)	46 (1.0)	137 (3.0)	19 (1.3)	177 (5.1)	327 (21.4)
	Unadjusted RR (95% CI)	ref	3.05 (2.05, 4.06)	1.34 (0.64, 2.04)	5.20 (3.54, 3.87)	21.84 (15.25, 28.43)
	Adjusted RR* (95% CI)	ref	3.68 (2.43, 4.93)	1.23 (0.58, 1.87)	5.72 (3.83, 7.61)	22.46 (15.57, 29.35)
ICU admission	n (col %)	286 (6.0)	522 (11.4)	169 (11.4)	733 (21.0)	771 (50.4)
	Unadjusted RR (95% CI)	ref	1.88 (1.62, 2.14)	1.87 (1.53, 2.21)	3.45 (3.00, 3.89)	8.29 (7.28, 9.30)
	Adjusted RR* (95% CI)	ref	2.36 (2.01, 2.70)	1.77 (1.44, 2.10)	4.01 (3.45, 4.57)	8.92 (7.73, 10.12)
In-hospital death	n (col %)	17 (0.4)	106 (2.3)	4 (0.3)	116 (3.3)	174 (11.4)

	Unadjusted RR (95% CI)	ref	6.33 (3.14, 9.52)	0.82 (-0.03, 1.67)	9.07 (4.53, 13.62)	31.01 (15.87, 46.15)
	Adjusted RR* (95% CI)	ref	5.17 (2.56, 7.79)	1.09 (-0.02, 2.19)	8.30 (4.14, 12.45)	30.82 (15.83, 45.81)

Logistic regression results were based on 45 imputed datasets.

*Risk ratios were adjusted for age, sex, race/ethnicity, receipt of influenza vaccine, influenza A subtype, and influenza season

Supplemental Figure 1. Proportions of specific respiratory exacerbations among adults hospitalised with laboratory-confirmed influenza and with specific underlying respiratory conditions by subgroup.

(A) Proportion with asthma exacerbation among patients with asthma by subgroup

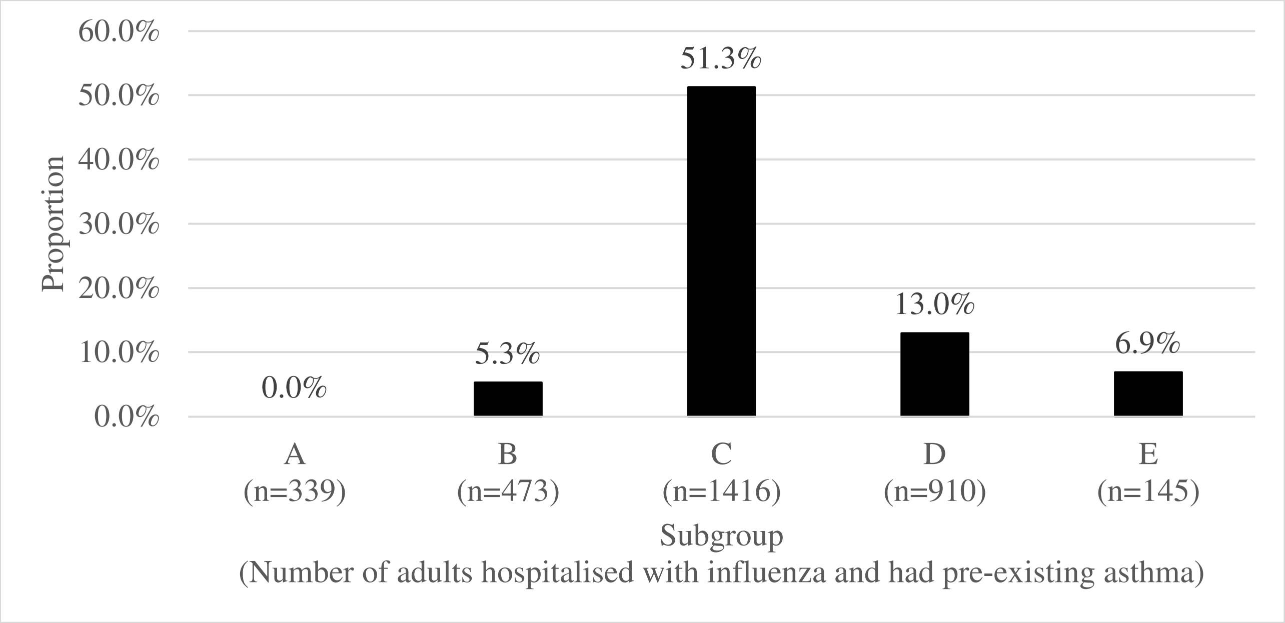

(B) Proportion with COPD exacerbation among patients with chronic lung disease by subgroup

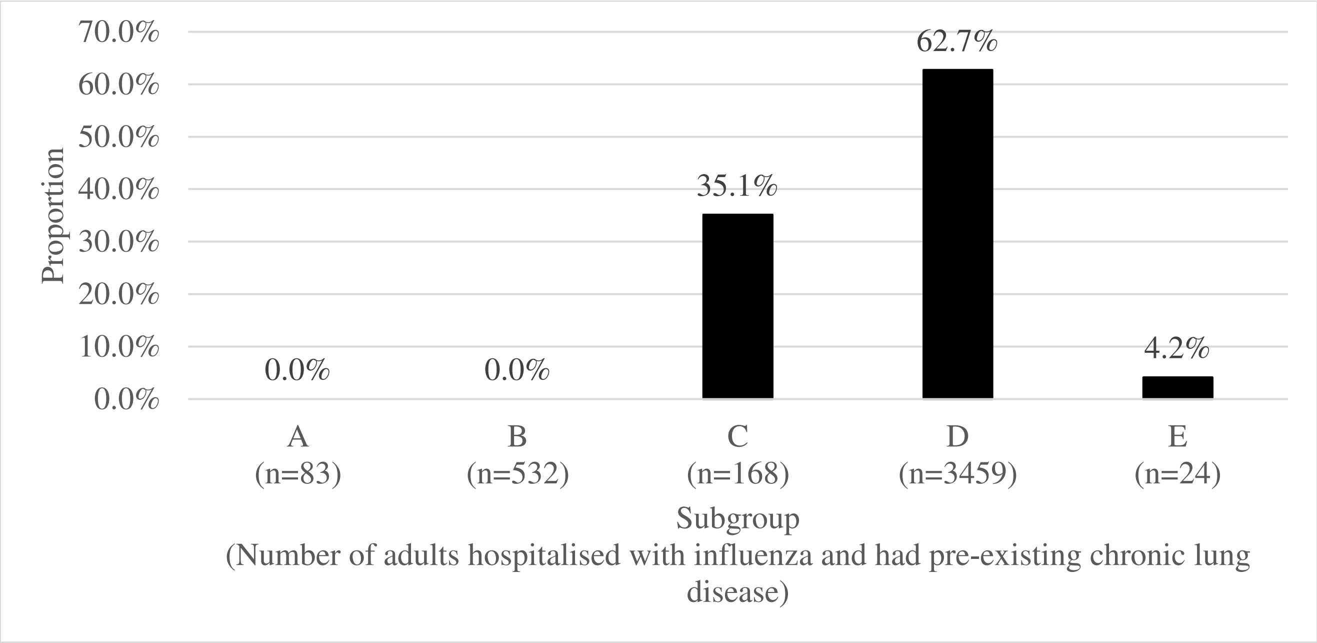

Supplemental Methods

Collection of clinical data on main case report form and supplemental disease severity form

Because of high counts of influenza-associated hospitalizations during the 2017-2018 and 2018-2019 seasons, an age- and site-stratified random sampling scheme was used to complete the main case report form for a representative sample of FluSurv-NET cases aged 50 years or older in 2017-2018 and adults aged 65 years or older in 2018-2019. Case report forms were completed for all patients aged 18-49 years. All in-hospital deaths were also sampled. Approximately half of the sites opted to implement a sampling strategy during each season.

For the supplemental disease severity form, sites selected a convenience sample among patients in their catchment area. For the 2017-18 season, three sites selected all cases admitted to all hospitals, five sites selected all cases from a subset of hospitals in their catchment area, two sites selected every fourth case admission to most or all hospitals in their catchment area, and three sites did not participate in the collection of this supplemental form[14].

Multiple imputation

Multiple imputation was performed in three steps. First, we imputed race/ethnicity and receipt of that season's influenza vaccine in the full data set. Secondly, since influenza B lineage was not completely captured among influenza B cases within FluSurv-NET and individuals with only influenza A had missing subtype, we imputed missing subtype in the subset of imputed data with the patients with influenza A only. Finally, we combined the data sets, and then derived a composite outcome variable and a three-categorical variable for the influenza type or subtype variable encompassing influenza A H3N2, A H1N1pdm09, and B viruses.
